# Error in Abstract and Visual Abstract

**DOI:** 10.1001/jamanetworkopen.2022.28862

**Published:** 2022-08-03

In the Original Investigation titled “Adjunctive Transdermal Cannabidiol for Adults With Focal Epilepsy: A Randomized Clinical Trial,”^1^ published July 8, 2022, there was an error in wording describing cannabidiol doses in the Abstract and the Visual Abstract. The text should have stated that patients received either 195 mg or 390 mg transdermal cannabidiol or placebo daily.^1^
